# Topotecan in a Real-World Small-Cell Lung Cancer Cohort: Prognostic Biomarkers Improve Selection of Patients for Second-Line Treatment

**DOI:** 10.3390/diagnostics14141572

**Published:** 2024-07-19

**Authors:** Laura Lambrecht, Paola Arnold, Jürgen Behr, Pontus Mertsch, Amanda Tufman, Diego Kauffmann-Guerrero

**Affiliations:** 1 Department of Medicine V, University Hospital, LMU Munich, 81337 Munich, Germany; laura.lambrecht@med.uni-muenchen.de (L.L.); paola.arnold@med.uni-muenchen.de (P.A.); juergen.behr@med.uni-muenchen.de (J.B.); pontus.mertsch@med.uni-muenchen.de (P.M.); amanda.tufman@med.uni-muenchen.de (A.T.); 2 Comprehensive Pneumology Center (CPC), Member of the German Center for Lung Research (DZL), University of Munich (LMU), 81337 Munich, Germany

**Keywords:** SCLC, topotecan, chemotherapy, prognostic markers

## Abstract

Background: Small-cell lung cancer (SCLC) is a highly aggressive tumor, and overall survival (OS) remains poor despite intensive efforts to develop new treatment strategies. In second line, topotecan is the only approved drug, with a median OS of 5.9 months. However, real-world SCLC patients are often in worse condition and harbor more comorbidities than study populations. Therefore, the real-world performance of topotecan may differ from that seen in studies. Here, we analyzed outcomes of SCLC patients receiving topotecan and identified predictive and prognostic markers. Patients and Methods: We retrospectively analyzed 44 consecutive SCLC patients receiving topotecan between 2015 and 2022. We analyzed baseline characteristics (age, ECOG-PS, topotecan cycles, and dosage) and pre-treatment blood values (LDH, CRP, sodium) as well as prognostic scores (neutrophil/lymphocyte ratio (NLR), thrombocyte/lymphocyte ratio (TLR), Glasgow Prognostic Score, prognostic nutritional score, systemic inflammation index (SII), and the prognostic index) extracted from electronic patients’ charts to identify predictive and prognostic markers. Results: In our cohort, mPFS and mOS were only 1.9 and 5.6 months, respectively. Gender, ECOG-PS, active brain metastases, NLR, GPS, PNI, and SII significantly influenced PFS and OS in univariate analysis. ECOG-PS (*p* > 0.001), active brain metastases (*p* = 0.001), and SII (*p* = 0.008) were significant independent prognostic variables in a multivariate COX regression model. Selecting patients by these three markers achieved an mPFS of 5.7 months and thus increased the mPFS three-fold. Patients not meeting all criteria had an mPFS of 1.8 months (*p* = 0.006). Patients identified by prognostic markers had an mOS of 9.1 months (*p* = 0.002). Conclusions: The efficacy of topotecan in SCLC real-world patients is poor, indicating that many patients were treated without any benefit. Easy-to-obtain markers can predict response and treatment efficacy and should therefore be validated in larger cohorts to identify patients who are more likely to benefit from topotecan.

## 1. Introduction

Small-cell lung cancer (SCLC) accounts for about 15% of all diagnosed lung cancers and is characterized by rapid and aggressive growth [1]. Despite good initial response to first-line chemotherapy, median survival of advanced SCLC patients remains poor, ranging from 7 to 10 months. The 1-year overall survival is only about 20–40% [1,2]. This is mainly due to early relapse after first-line therapy and lack of effective therapies in further treatment lines [3].

Topotecan is the only FDA- and EMA-approved second-line treatment in SCLC based on the observation that topotecan versus cyclophosphamide, doxorubicin, and vincristine (CAV) in patients relapsing at least 60 days after first-line treatment resulted in a numerical higher response rate of 24.3% (topotecan) compared to 18.3% (CAV). Median overall survival (OS) was 5.7 months in both groups, but the topotecan group achieved a more favorable toxicity profile [4]. O’Brien et al. compared oral topotecan versus best supportive care (BSC) in patients with relapsed SCLC not considered for standard intravenous therapy and found a significantly longer overall survival in the topotecan group with median survival times of 25.9 weeks compared to 13.9 weeks in the BSC group [5]. Since this time, despite many clinical trials evaluating promising agents, no further second-line treatment achieved approval for SCLC patients in Europe. 

However, topotecan is associated with relevant, especially hematological side effects, and most patients do no benefit from this treatment [6]. Furthermore, real-world SCLC patients are mostly in worse condition and harbor more comorbidities than study populations. Therefore, one has to fear that the small survival advantage of topotecan might vanish in a real-world cohort. Furthermore, although topotecan is the only approved drug for the second-line treatment of patients with SCLC, we still have no verified markers to forecast the tolerance and effectiveness of the treatment in individual patients.

Reliable predictive and prognostic markers are therefore needed to select patients with higher probability for response to topotecan. For this reason, this study aims at investigating outcomes of real-world SCLC patients receiving topotecan and tries to identify predictive and prognostic markers.

## 2. Materials and Methods

### 2.1. Study Population 

We identified all patients with SCLC treated with topotecan between 2015 and 2022 at our tertiary care lung cancer center. Patients were identified using our electronic cancer documentation system and our pharmacy delivery repository searching for all patients documented with SCLC of the lung receiving topotecan. Results have been verified by pathological reports and clinical documentation. Patient characteristics in terms of gender, age, performance status, tumor stage, smoking status, information on treatment modalities, and dosage were collected from patient charts (paper-based and electronic records). Age was calculated at the time of diagnosis. Treatment response was evaluated by CT- or PET-CT-based monitoring using the Response Evaluation Criteria in Solid Tumors 1.1 (RECIST) [7]. We did not use iRECIST criteria in patients receiving immunotherapy treatment before topotecan because of relevant varying times since immuno-oncology teratments (IO). Progression-free survival (PFS) was calculated from the date of first topotecan dose until confirmed disease progression or death. Overall survival (OS) was calculated from the date of first topotecan dose until death. Pre treatment blood values were extracted from the electronic patients’ charts. The time frame in which the values have to be recorded was between 0–10 days before treatment start. 

### 2.2. Statistical Analyses 

For data description, mean values and standard deviations were used. Comparisons between groups were performed by the Mann–Whitney-U test, or by chi-square-tests in case of categorical variables. Estimation of progression-free survival (PFS) and overall survival (OS) was carried out using the Kaplan–Meier method with the logrank test. For multivariate survival analysis, COX-regression models were used, including variables with significant results in the univariate analysis. Cut-offs for metric valiables are set by the upper or lower reference value of our laboratory for clinical chemistry (blood values) or by cut-off from the literature or our own publications (calculated values) [8,9]. The level of statistical significance was determined at *p* < 0.05. All statistical analyses were performed using SPSS 29 statistical software (IBM Corp., Armonk, NY, USA). 

## 3. Results

We analyzed all patients with SCLC receiving topotecan between 2015 und 2022. The cohort showed a light male predominance (56.8%), and the mean age was 64.3 years. All patients had stage IV disease confirmed by CT or PET-CT scans before treatment start with topotecan and were active or former smokers. A total of 43.2% harbored reduced performance status (ECOG-PS 2 or higher), and 34.1% had active brain metastases at the start of topotecan. About a third were pretreated with chemo-immunotherapy (atezolizumab) and 29.5% received radio-chemotherapy (RCT) in first-line treatment. These patients received either concurrent or sequential RCT in a limited stage as a first-line treatment. Only two patients received thoracic consolidative radiotherapy after systemic treatment in stage IV. Those are listed under chemo(-immune) therapy. Table 1 summarizes baseline characteristics of the study cohort.

### 3.1. Survival Analysis of the Whole Cohort

**Figure 1:**
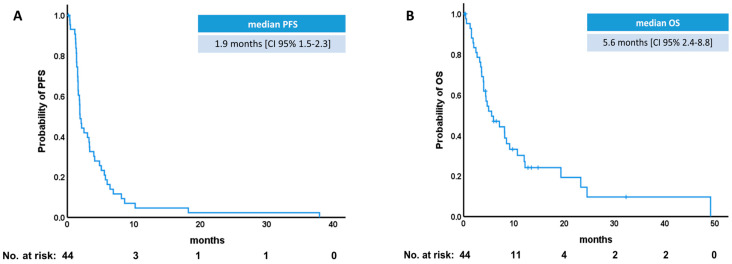
Survival curves of the study cohort. (**A**) Progression-free survival (PFS). (**B**) Overall survival (OS).

The median PFS of topotecan treatment in the whole cohort was only 1.9 months [CI 95% 1.5–2.3] (Figure 1A), while overall survival was 5.6 months [CI 95% 2.4–8.8] (Figure 1B). Overall response rate (ORR) was 13.6% with a disease control rate (DCR) of 31.8%. The median duration of response (mDoR) was 5.1 months [CI 95% 3.7–6.7].

**Figure 2:**
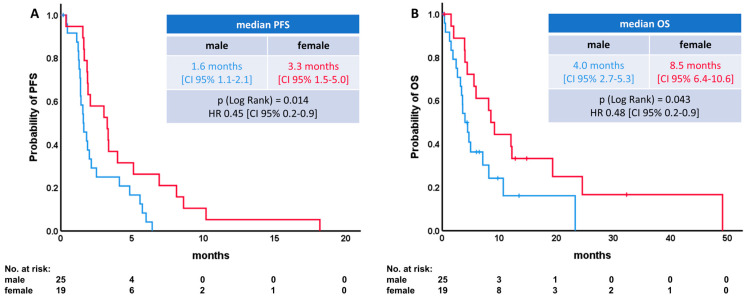
Survival differences between male and female patients. (**A**) Progression-free survival (PFS). (**B**) Overall survival (OS).

Interestingly, women showed significantly improved median PFS (3.3 vs. 1.9 months) and median OS (8.5 vs. 4.0 months) compared to male patients (Figure 2A,B).

**Figure 3:**
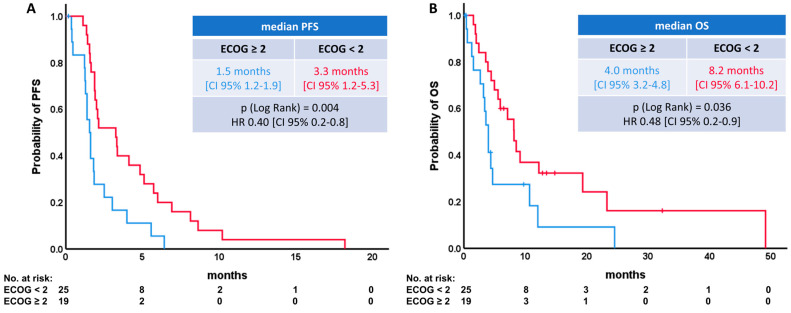
Survival differences stratified by ECOG-Performance status. (**A**) Progression-free survival (PFS). (**B**) Overall survival (OS).

As expected, ECOG performance status (PS) did significantly influence PFS and OS, showing that patients with ECOG-PS of 2 or higher had no meaningful benefit from topotecan treatment (Figure 3A,B). Moreover, older patients (≥70 years) had no reduced survival per se.

Time from first diagnosis of SCLC until topotecan treatment and treatment-free interval (TFI) before topotecan did not affect survival after topotecan treatment as well as prior treatment with atezolizumab. Furthermore, mean topotecan dosage did not change PFS, but, interestingly, patients with lower mean dosages (<1.25 mg/m^2^) had numerically longer OS. Patients without active brain metastases at the beginning of topotecan treatment had significantly longer PFS, which did not translate into an OS advantage. On the other hand, patients with and without prior WBRT did not differ regarding their survival. First-line treatment, sole systemic therapy, or radio-chemotherapy (RCT) had no significant impact on survival as well as type of systemic first-line treatment (with or without IO). However, patients with initial RCT had a meaningful longer OS with 12.0 months compared to patients with systemic treatment (Table 2).

### 3.2. Toxicity

As expected, hematological side effects were common. A total of 88% of all patients had any grade of anemia, with grade 3 and 4 accounting for 45.5% and 4.5%, respectively. A total of 65.9% had thrombocytopenia (grade 3: 22.7% and grade 4: 27.3%). Leucopenia was observed in 66% of the patients (grade 3: 22.7% and grade 4: 22.7%).

### 3.3. Prediction of Response

Higher topotecan dosage (≥1.25 mg/m^2^) was significantly associated with better response to treatment (*p* = 0.010). Besides this, we found no further predictive markers for treatment response for either categorial or for continuous variables in our cohort (Table 3 and Table 4).

### 3.4. Prognostic Value of Different Markers and Scores

Elevated pre-therapeutic LDH levels were significantly associated with poorer PFS, OS was prolonged by trend, but not statistically significant in patients with normal LDH values. CRP values did not significantly correlate with PFS or OS. However, we found a neutrophil-to-lymphocyte ratio (NLR) with a cut-off of 6 significantly affecting PFS and OS of topotecan-treated patients. Low sodium values were associated with reduced OS but showed no significant impact on PFS. Low thrombocyte-to-lymphocyte ratio (TLR) values were revealed to be associated with significantly longer PFS but did not translate to longer OS. A Glasgow Prognostic Score (GPS) score of 0 was strongly correlated with better OS and PFS. Elevated SII was significantly associated with worse PFS. The prognostic index (PI) differed highly significantly in patients regarding both PFS and OS. Results of univariate survival analyses are shown in Table 4.

### 3.5. Multivariate Analysis

**Figure 4:**
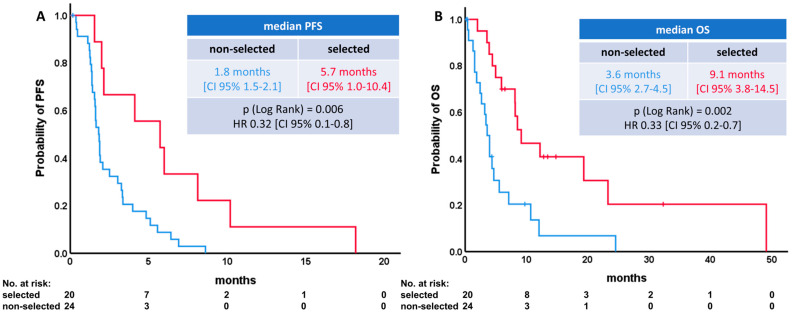
Survival analysis after patient selection based on the examined prediction models. (**A**) Progression-free survival (PFS). (**B**) Overall survival (OS).

Integrating all significant variables from the univariate analysis in a multivariate COX regression model for PFS revealed ECOG-PS (*p* < 0.001), active brain metastases (*p* = 0.001), and SII (*p* = 0.008) as significant independent prognostic markers. Selecting patients by these three markers achieved a PFS of 5.7 months [CI 95% 2.3–10.4] and thus increased the PFS of the total cohort three-fold. Patients not meeting all criteria had a PFS of 1.8 months [CI 95% 1.5–2.1] (Figure 4A). For OS, multivariate analysis also revealed ECOG-PS (*p* < 0.001) and PNI (*p* < 0.001) as independent prognostic markers. Selecting patients by these markers achieved a median OS of 9.1 months [CI 95% 3.8.–14.5] and thus increased the OS of the total cohort 1.6-fold. Patients not meeting all criteria had a median PFS of 3.6 months [CI 95% 2.7–4.5] (Figure 4B).

## 4. Discussion

In this retrospective single-center analysis, we sought to analyze efficacy of topotecan second-line treatment in unselected SCLC patients and identify predictive and prognostic markers for improved patient selection.

Efficacy of topotecan second-line treatment in SCLC have been examined in some phase II and phase III studies. In a first phase II trial, Perez-Soler et al. showed a modest effect of topotecan in pretreated SCLC patients with a response rate of 11%. The duration of response recached from 7 to 19 weeks, and the median survival was 4.6 months [10]. A larger phase II trial of second-line topotecan with 92 patients was published one year later. The authors found a response rate of only 6.4% in platinum refractory patients, but of 37.8% in sensitive disease, respectively. The median survival was 4.7. months in refractory and 6.9 months in sensitive patients [11]. The first randomized second-line trial published by von Pawel et al. tested topotecan versus cyclophosphamide, doxorubicin, and vincristine (CAV) in patients relapsing at least 60 days after first-line treatment. Response rates were 24.3% (Topotecan) and 18.3% (CAV). Median PFS and median OS (5.7 months in both groups) were not significantly different, but the topotecan group achieved a more favorable toxicity profile [4]. A Japanese phase II trial of second-line topotecan in relapsed SCLC showed a response rate of 26% an OS of 8.6 months [12]. In our cohort, the response rate was 13.6%, median PFS was 1.9 months, and median OS was 5.6 months and thus are in line with survival results found in the pivotal studies.

We found a significantly improved PFS and OS in female patients. Looking deeper into baseline characteristics, it showed that women received more WBRT often and are often initially treated with chemoradiotherapy.

Nearly 10% of our patients have been treated with oral topotecan. We found no difference of efficacy compared to intravenous therapy. This confirmed the results of two earlier studies and indicates that a more frequent use of oral topotecan should be considered to improve patients’ comfort [13,14]. Besides this, in our cohort, lower topotecan dosage seemed not to impact PFS with numerically increased OS, although response rates were increased in higher topotecan dosages. This might be attributed to the relevant increased toxicity with higher doses of topotecan. Moreover, the drug leads to higher response rates, but limits survival by treatment-associated complications. Furthermore, we found that age per se does not impair effectiveness of topotecan treatment. This is in line with a previous study showing that topotecan monotherapy resulted in more toxicity in elderly patients, but was as effective as in younger patients [15].

We identified the ECOG-PS, active brain metastases, and the systemic inflammation index (SII) as potent selection parameters to identify patients with better PFS. By selecting patients with these three markers, the PFS could be increased three-fold in our cohort.

For OS selecting patients by ECOG-PS and prognostic nutritional index (PNI), we found a significant increase of 1.6-fold in survival compared with unselected patients.

Other studies support these markers. Treat and colleagues demonstrated in a pooled analysis of 480 SCLC patients treated with second-line topotecan that patients’ performance status is associated with overall survival [16]. Another meta-analysis showed that improvement of PS is a reliable predictor of response to topotecan treatment [17].

Topotecan can cross the blood–brain barrier. But although a phase II study showed an intracranial response rate of 33% in pretreated patients, the OS remains at only 3.6 months in this analysis [18]. Another small study did not show a meaningful intracranial activity of topotecan in SCLC [19], underlying the negative effect of brain metastases we also found in our cohort.

Taking these results together, you should assume that topotecan as second-line treatment in an unselected real-world population might not be beneficial, especially in light of high toxicity rates. Therefore, patient selection, for example using the easy-to-obtain markers we examined in this study, might increase treatment efficacy and prevent unnecessary toxicity. Furthermore, other more effective second-line therapy options are urgently needed as more aggressive treatment regimens by combining topotecan with other chemotherapeutic drugs as cisplatin or etoposide resulted in no or only minimal increases in overall survival but relevant more toxicity [20,21,22,23,24,25]. Also, the combination of biologicals with topotecan as Bcl2-Inhibotors or VEGF-Inhibitors did not improve efficacy in this setting [26,27,28]. Amrubicin demonstrated possible superiority over topotecan in the second-line setting in phase II studies [29,30]. Approved in Japan, amrubicin is not approved in Europe and the US due to a negative phase III study [31]. Also, the combination of amrubicin and topotecan showed promising efficacy, but was not investigated further [32].

Our study harbors some limitations. The first is the small size of the cohort and the retrospective character of the study. However, as we included all consecutive SCLC patients treated with second-line topotecan and did not exclude patients from the analysis, we analyzed a true real-world cohort and our results may help to guide treatment decisions in second-line to identify patients who are more likely to benefit from topotecan. Furthermore, given the low efficacy of the topotecan treatment in combination with high toxicity, patients should, whenever possible, be enrolled in clinical studies helping to identify more potent therapy options in this setting.

## 5. Conclusions

The efficacy of topotecan in SCLC real-world patients is poor, indicating that many patients were treated without any benefit. The analysis of our cohort revealed easy-to-obtain markers (ECOG-PS, active brain metastases, and the systemic inflammation index (SII) for PFS as well as ECOG-PS and prognostic nutritional index (PNI) for OPS) to predict response and treatment efficacy. These markers should therefore be validated in larger cohorts to identify patients more likely to benefit from topotecan.

## Figures and Tables

**Table 1:** Baseline characteristics of the study cohort. ECOG-PS (Eastern Cooperative Oncology Group Performance Status), IO (immune oncology), WBRT (whole-brain radiation therapy), Sdev (standard deviation), PR (partial response), SD (stable disease), PD (progressive disease).

	*n* = 44
Male (*n* (%))	25 (56.8%)
Female (*n* (%))	19 (43.2%)
Age (mean ± Sdev)	64.3 ± 9.9
Males	65.52 ± 10.4
Females	62.6 ± 9.2
ECOG-PS	
0	6 (13.6%)
1	19 (43.2%)
2	14 (31.8%)
3	5 (11.4%)
Smoking status (*n* (%))	
Current smoker	9 (20.5%)
Former smoker	35 (79.5%)
Pharmaceutical form (*n* (%))	
Intravenous	40 (90.9%)
Oral	4 (9.1%)
Median of applied cycles [range]	3 (1–17)
Mean applied dosage [range]	1.22 mg/m^2^ [0.625–2.4 mg/m^2^]
Median time from first diagnosis until topotecan	8.3 months
Median time from last treatment until topotecant (treatment free intervall, TFI)	1.7 months
<3 months (*n* (%))	32 (72.7)
≥3 months	12 (27.3)
Prior IO treatment (*n* (%))	
Yes	15 (34.1%)
No	29 (65.9%)
Active brain metastases (*n* (%))	
Yes	15 (34.1%)
No	29 (64.9%)
WBRT before topotecan (*n* (%))	
Yes	20 (45.5%)
No	24 (54.5%)
1st-line treatment (*n* (%))	
Chemo(-immuno) therapy	31 (70.5%)
Radiochemotherapy	13 (29.5%)
Best response to topotecan treatment (*n* (%))	
PR	6 (13.6%)
SD	8 (18.2%)
PD	23 (52.3%)
Death	7 (15.9%)

**Table 2:** Univariate survival analyses of the study cohort stratified by baseline characteristics. PFS (progression-free survival) in months, OS (overall survival) in months, HR (hazard ratio), CI (confidence interval).

	Median PFS [CI 95%]	HR [CI 95%]	*p*-Value [Log Rank]	Median OS [CI 95%]	HR [CI 95%]	*p*-Value [Log Rank]
Gender						
Male	1.6 [1.1–2.1]		*p* = 0.014	4.0 [2.7–5.3]		*p* = 0.043
Female	3.3 [1.5–5.0]	0.45 [0.2–0.9]		8.5 [6.4–10.6]	0.48 [0.2–0.9]	
ECOG						
<2	3.3 [1.2–5.3]	0.40 [0.2–0.8]	*p* = 0.004	8.2 [6.1–10.2]	0.48 [0.2–0.9]	*p* = 0.036
≥2	1.5 [1.2–1.9]			4.0 [3.2–4.8]		
Age						
<70 years	1.9 [0.4–3.5]		*p* = 0.433	5.9 [2.0–9.8]		*p* = 0.713
≥70 years	1.9 [1.5–2.3]	1.2 [0.6–2.6]		4.0 [3.4–4.6]	1.1 [0.5–2.6]	
Time until topotecan						
≤8.3 months	1.9 [1.5–2.3]		*p* = 0.719	4.4 [3.6–5.1]		*p* = 0.484
>8.3 months	1.9 [0.3–3.5]	0.66 [0.3–1.3]		8.6 [4.4–12.7]	0.78 [0.4–1.6]	
Median time from last treatment until topotecant (treatment free interval, TFI)						
<3 months (*n* (%))	1.9 [1.6–2.2]	1.9 [0.6–5.8]	*p* = 0.205	5.0 [3.4–6.5]	1.5 [0.1–2.5]	*p* = 0.711
≥3 months	1.9 [0.0–5.8]			9.1 [1.7–16.6]		
Mean topotecan dosage						
<1.25 mg/m^2^	3.3 [1.2–5.3]		*p* = 0.547	8.1 [3.9–12.3]		*p* = 0.111
≥1.25 mg/m^2^	1.6 [1.2–2.0]	1.4 [0.8–2.7]		4.3 [3.1–5.7]	1,7 [0.9–3.5]	
Prior IO treatment						
Yes	1.8 [1.2–2.4]		*p* = 0.903	5.0 [0.8–9.1]		*p* = 0.855
No	2.1 [0.4–3.9]	0.9 [0.5–1.6]		5.9 [0.5–11.4]	2.1 [0.5–8.8]	
Active brain metastases						
No	3.0 [1.5–4.6]		*p* = 0.028	7.1 [2.5–11.7]		*p* = 0.633
Yes	1.6 [1.3–1.9]	1.9 [1.0–3.6]		4.7 [3.7–5.7]	1.2 [0.6–2.6]	
WBRT before topotecan						
No	2.1 [0.0–4.4]		*p* = 0.475	7.1 [2.5–11.7]		*p* = 0.571
Yes	1.8 [1.7–2.0]	1.1 [0.6–2.0]		5.0 [2.7–7.2]	0.8 [0.4–1.6]	
1st-line treatment						
Chemo(-immuno) therapy	1.8 [0.9–2.8]		*p* = 0.638	4.4 [3.6–5.2]		*p* = 0.089
Radiochemotherapy	1.9 [1.5–2.4]	0.7 [0.3–1.4]		12.0 [6.8–17.2]	0.5 [0.2–1.1]	

**Table 3:** Categorial and continuous variables tested for impact on response of topotecan treatment.

**Categorial Variables**			
**Variable**	***p*-Value**	**Variable**	***p*-Value**
Gender (male vs. women)	*p* = 0.054	Active brain metastases (yes vs. no)	*p* = 0.598
ECOG (0 vs. 1 vs. 2 vs. 3)	*p* = 0.085	WBRT (yes vs. no)	*p* = 0.813
Application (p.o. vs. i.v.)	*p* = 0.152	1st-line treatment (CX vs. RCT)	*p* = 0.420
GPS (0 vs. 1 vs. 2)	*p* = 0.207	Age (<70 vs. ≥70)	*p* = 0.709
Prior IO treatment (yes vs. no)	*p* = 0.128	Time till Topotecan (≤8.3 vs. >8.3 months)	*p* = 0.195
Dosage (<1.25 mg/m^2^ vs. ≥1.25 mg/m^2^)	*p* = 0.010	LDH (≤250 vs. >250 U/l)	*p* = 0.195
CRP (≤0.5 vs. >0.5 mg/dL	*p* = 0.32	NLR (≤6 vs. >6)	*p* = 0.598
Sodium (<135 vs. ≥135 mmol/L)	*p* =0.092	TLR (≤200 vs. >200)	*p* = 1
PNI (<40 vs. ≥40)	*p* = 0.262	SII (<1500 vs. ≥1500)	*p* = 0.349
PI (1 + 2 vs. 3 + 4)	*p* = 0.287		
**Continuous Variables**			
**Variable**	***p*-Value**	**Variable**	***p*-Value**
Age	*p* = 0.830	NLR	*p* = 0.257
Mean topotecan dosage	*p* = 0.009	TLR	*p* = 0.860
LDH	*p* = 0.059	PNI	*p* = 0.174
CRP	*p* = 0.007	SII	*p* = 0.166
Sodium	*p* = 0.419		

**Table 4:** Prognostic impact on survival of examined markers and scores. PFS (progression-free survival) in months, OS (overall survival) in months, HR (hazard ratio), CI (confidence interval).

	Median PFS [CI 95%]	HR [CI 95%]	*p*-Value [Log Rank]	Median OS [CI 95%]	HR [CI 95%]	*p*-Value [Log Rank]
LDH						
≤250 U/L	3.0 [0.9–5.1]		*p* = 0.006	8.5 [4.0–13.1]		*p* = 0.370
>250 U/L	1.6 [1.2–2.1]	2.2 [1.2–4.3]		3.6 [1.8–5.4]	1.4 [0.7–2.8]	
CRP						
≤0.5 mg/dL	3.4 [0.7–6.0]		*p* = 0.089	9.1 [5.7–12.6]		*p* = 0.102
>0.5 mg/dL	1.6 [1.3–2.0]	1.6 [0.8–3.1]		4.4 [2.7–6.0]	1.6. [0.8–3.1]	
NLR						
≤6	3.0 [0.9–5.1]		*p* = 0.002	8.2 [4.1–12.2]		*p* = 0.01
>6	1.3 [1.1–1.6]	2.5 [1.3–4.9]		4.4 [1.8–7.1]	2.6 [1.2–5.5]	
Sodium						
<135 mmol/L	1.6 [1.5–1.7]		*p* = 0.066	4.4 [2.9–5.8]		*p* = 0.029
≥135 mmol/L	3.0 [1.6–4.4]	0.5 [0.3–1.1]		8.1 [4.1–12.2]	0.4 [0.2–0.9]	
TLR						
≤200	3.0 [0.4–5.7]		*p* = 0.020	8.1 [3.0–13.2]		*p* = 0.734
>200	1.9 [1.5–2.3]	1.9 [1.0–3.7]		4.7 [3.5–5.8]	1.1 [0.6–2.2]	
GPS						
0	3.0 [0.4–5.6]		*p* = 0.011	9.1 [5.6–12.66]		*p* = 0.008
≥1	1.6 [1.2–2.1]	2.3 [1.2–4.6]		4.0 [2.2–5.7]	2.6 [1.3–5.4]	
PNI						
<40	1.4 [1.2–1.7]		*p* = 0.015	2.4 [1.2–3.6]		*p* < 0.001
≥40	2.5 [0.9–4.1]	0.4 [0.2–0.9]		8.6 [2.4–8.8]	0.2 [0.1–0.4]	
SII						
<1500	3.0 [0.9–5.1]		*p* = 0.014	8.1 [3.0–13.3]		*p* = 0.431
≥1500	1.6 [1.3–1.8]	2.1 [1.1–3.9]		4.7 [2.9–6.5]	1.3 [0.7–2.7]	
PI						
1–2	3.3 [2.6–4.0]		*p* = 0.003	9.1 [2.4–15.9]		*p* = 0.001
3–4	1.6 [1.3–1.9]	2.4 [1.3–4.7]		3.6 [2.9–4.2]	3.2 [1.5–6.6]	

## Data Availability

The authors state to share the original data on reasonable request.
